# The Rare Variant rs35356162 in *UHRF1BP1* Increases Bladder Cancer Risk in Han Chinese Population

**DOI:** 10.3389/fonc.2020.00134

**Published:** 2020-02-11

**Authors:** Junlong Wu, Meilin Wang, Haitao Chen, Jianfeng Xu, Guiming Zhang, Chengyuan Gu, Qiang Ding, Qingyi Wei, Yao Zhu, Dingwei Ye

**Affiliations:** ^1^Department of Urology, Fudan University Shanghai Cancer Center, Shanghai, China; ^2^Department of Oncology, Shanghai Medical College, Fudan University, Shanghai, China; ^3^Department of Genetic Toxicology, The Key Laboratory of Modern Toxicology of Ministry of Education, School of Public Health, Nanjing Medical University, Nanjing, China; ^4^State Key Laboratory of Organ Failure Research, Guangdong Key Laboratory of Viral Hepatitis Research, Department of Infectious Diseases and Hepatology Unit, Nanfang Hospital, Southern Medical University, Guangzhou, China; ^5^Fudan Institute of Urology, Huashan Hospital, Fudan University, Shanghai, China; ^6^Center for Cancer Genomics, Wake Forest School of Medicine, Winston-Salem, NC, United States; ^7^Department of Urology, The Affiliated Hospital of Qingdao University, Qingdao, China; ^8^Cancer Institute, Fudan University Shanghai Cancer Center, Shanghai, China; ^9^Duke Cancer Institute, Duke University Medical Center, Durham, NC, United States

**Keywords:** bladder cancer, *UHRF1BP1*, exome array, Chinese, single nucleotide variant

## Abstract

**Background:** Seventeen loci have been found to be associated with bladder cancer risk by genome-wide association studies (GWAS) in European population. However, little is known about contribution of low-frequency and rare variants to bladder cancer susceptibility, especially in Eastern population.

**Methods:** We performed a three-stage case-control study including 3,399 bladder cancer patients and 4,647 controls to identify low-frequency and rare variants associated with bladder cancer risk in Han Chinese. We examined exome-array data in 1,019 bladder cancer patients and 1,008 controls in discovery stage. Two replication stages were included to validate variants identified. Bonferroni adjustment was performed to define statistical significance. Logistic regression was conducted to evaluate single marker association with bladder cancer risk. We used SKAT-O method to perform gene level-based analysis. We also conduct additional experiments to explore the underlying mechanism of filtered gene(s).

**Results:** We identified a novel rare coding variant (rs35356162 in *UHRF1BP1*: G > T, OR = 4.332, *P* = 3.62E-07 < 7.93E-07, Bonferroni cutoff) that increased bladder cancer risk in Han Chinese. Gene-level analysis showed a significant association of *UHRF1BP1* (*P* = 4.47E-03) with bladder cancer risk. Experiments indicated down-regulation of *UHRF1BP1* promoted migration and invasion through epithelial-mesenchymal transition in bladder cancer cell lines.

**Conclusion:** The rare variant of *UHRF1BP1*, rs35356162, increases bladder cancer risk in Han Chinese and UHRF1BP1 might act as a tumor suppressor in bladder cancer development and progression.

**Summary:** Little is known about potential contribution of low-frequency and rare variants to bladder cancer susceptibility. We performed a three-stage case-control study and identified a new rare variant, rs35356162 in *UHRF1BP1*, which increased bladder cancer risk in Han Chinese.

## Introduction

Bladder cancer is the 7th most common cancer globally (1) and ranks 1st in urologic malignancies in China (2). Bladder cancer incidence varies among different geographic regions, owing to different genetic background, lifestyles and environmental factors (3–5). Cigarette smoking and occupational exposure to aromatic amine compounds are two well-known risk factors (6), while genetic predisposition factors may explain one-third of all the bladder cancer cases (7).

Genome-wide association studies (GWAS) have identified 17 independent loci and single nucleotide variants (SNVs) that contribute to bladder cancer susceptibility in European population [(8–14); Table S1]. Many loci have been replicated in additional studies, and additional new loci found to be, associated with bladder cancer risk in Chinese population (15–21). GWA studies have been successful in identifying common variants involved in complex trait etiology. However, SNVs identified by GWAS are common variants with a minor allele frequency (MAF) over 5%, which only had small individual effect sizes with an odds ratio (OR) ranging from 1.1 to 1.5 mostly. Thus, the “missing heritability” remains an issue in interpreting GWAS results (22). It was reported that < 10% of genetic variance could be explained by common variants via GWAS for the majority of complex traits (23). Additionally, low-frequency (1% ≤ MAF ≤ 5%) and rare (MAF < 1%) variants were not included in modern GWAS chips, which could lead to missing heritability as well.

To date, many low-frequency and rare variants affecting the risk of complex traits have been found, such as rare coding mutations of *BRIP1* and *RAD51D* in ovarian cancer (24, 25), and rare variants of *ATM, RAD50*, and *PALB2* in breast cancer (26). However, little is known about the contribution of low-frequency and rare variants to bladder cancer risk. Therefore, we performed a three-stage study, aiming to identify new low-frequency or rare variants that are associated with bladder cancer risk in Han Chinese. Functions of the relevant gene in bladder cancer were also explored.

## Materials and Methods

### Study Population and Design

This study is a three-stage case-control study. The discovery stage included 1,019 bladder cancer cases and 1,008 controls to filter variants associated with BCa risk. In replication I and II stages, a total of 2,404 BCa cases and 3,639 controls were recruited to validate variants accordingly. Detailed recruitment criteria and study design supplements was summarized in Supplementary Materials and Methods. All individuals recruited in this study were unrelated Han Chinese people.

Slides of BCa cases were confirmed by two pathologists independently and results were reported based on the 2004 WHO/ISUP classification criteria. Clinical characteristics were collected via medical records. Cancer history, symptom and smoking status was collected from medical records or phone inquiry. A smoking status of “Yes” represented current smokers at diagnosis or those who had ever smoked daily for over 1 year before diagnosis.

This study was performed according to the ethical standards of the Helsinki Declaration II and approved by the Scientific and Ethical Committee of Fudan University Shanghai Cancer Center and other Institutional Review Board of participating hospitals. Informed consents were obtained from all subjects.

### Exome Array Genotyping and Calling

We performed exome array genotyping using Illumina HumanExome-12 v1.1 beadchip (see URL: Exome Chip Wiki) in 1,019 bladder cancer cases and 1,008 controls in the discovery stage. Genotype calling was carried out by standard Illumina's GenTrain version 2.0 clustering algorithm using GenomeStudio software (V2011.1). Cluster boundaries were determined using Illumina's standard cluster file. The datasets used and/or analyzed during the current study are available at https://doi.org/10.17632/bkvnsfgd4y.1.

### Exome Chip Analysis

To select proper SNVs for further analysis, we conducted quality control of samples and SNPs according to the procedures described in Supplementary Materials and Methods. Finally, 20 cases failed IBS analysis (Figure S1), 3 cases were duplicated samples and 1 case had incomplete clinical data. Notably, 636 SNVs failed HWE test in control group. So after quality control of samples and SNVs, a total of 995 cases and 1,008 controls with 63,047 SNVs remained (Details of quality control in Tables S2, S3). Because we shared the same controls with ChinaPCa project, these 1,008 controls survived same filtering procedures before (27). Principal component analysis (PCA) was performed using EIGENSOFT. A set of SNVs that showed low linkage disequilibrium (LD; *r*^2^ < 0.1) were used to estimate population outliers in a principal component analysis. The result was shown in Figure S2.

### Selection of Variants in Replication Stages

SNVs detected in discovery stage were classified into three categories: common variants, low-frequency, and rare variants, and reported variants previously. These three kinds of variants were filtered following different procedures (Tables S4–S6). *P-*value thresholds for selection were presented as follows: 0.001 for common variants, 0.01 for low-frequency and rare variants and 0.05 for variants in previous reported GWAS data. Twenty-six SNVs were selected for validation in replication I stage using Sequenom MassARRAY. Based on the combined results from discovery stage and replication I stage, validation was performed in replication II stage using TaqMan probes (Life Technology, Carlsbad, CA, USA). Results were analyzed using SDS2.4 software (Applied Biosystems, Foster City, CA, USA). All genotyping was conducted independently by technicians in a blinded manner. Detailed filtering procedures were shown in Supplementary Materials and Methods.

### Functional Experiments

Additional experiments were performed to explore functions of certain gene(s) selected from three-stage study in bladder cancer development and progression. Detailed description of cell lines and culture, plasmids construction and lentivirus preparation, RNA extraction, reverse transcription and quantitative real-time PCR analysis, antibodies for western blot, cell cycle assay, cell proliferation, migration and invasion assays were presented in Supplementary Materials and Methods.

### Statistical Analysis

We performed univariate logistic regression analysis without adjustments of clinical features to calculate odds ratio (OR) and 95% confidence interval (CI) to estimate association between single variant and bladder cancer risk in an additive model. If no polymorphism was detected in control group, single-variant association analysis was performed using Fisher exact test. Hardy-Weinberg Equilibrium was compared using Pearson's Chi-square test. An identity-by-state similarity score was obtained using PLINK (see URLs). For gene-level analysis, we conducted the sequence kernel association optimal (SKAT-O) test, using reference Gene file from UCSC. The SKAT-O test included all the SNVs which survived filtering procedures, 63,047 SNVs in total. Statistical analysis and plotting were mainly carried out using R software (see URLs) and PLINK. In addition, Bonferroni adjustment was used for three stages combined to find out significant variant which was associated with bladder cancer risk. The Bonferroni cutoff was calculated as 0.05/63,047 SNVs, which meant the significance boundary was 7.93E-07.

PolyPhen2 (see URLs) was used to predict the functional impact of certain variant based on sequence and structure's predictive methods. Amino acid conservation analysis was based on multiple sequences alignment performed on Vector NTI 11.5.1 (Invitrogen, Carlsbad, CA, USA) and was plotted using CTex (see URLs).

## Results

### Exome Array Analysis

**Figure 1:**
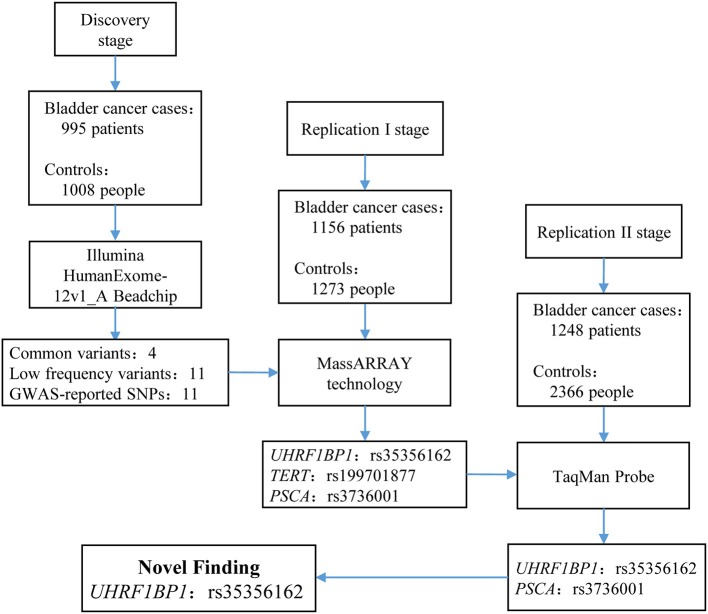
Flow chart of study design and summary of the three-stage study. In discovery stage, we enrolled 995 bladder cancer cases and 1,008 controls to perform exome chip analysis. After filtering, 26 SNVs remained and entered into replication I stage for further validation via MassARRAY technology. In replication I stage, only 3 SNPs survived. These three SNPs entered into replication II stage for validation. Finally, only rs35356162 in *UHRF1BP1* and rs3736001 in *PSCA* survived. However, only rs35356162 in *UHRF1BP1* achieved statistical significance after Bonferroni adjustment. More study design supplements and aim of each stage can be found in Supplementary Materials and Methods.

Demographics of the participants in this three-stage study are shown in Table 1. The flow chart of our study design and primary results are summarized in Figure 1.

**Table 1:** Characteristics of subjects analyzed in the discovery and subsequent replication stages.

**Characteristics**	**No. (%)**					
	**Discovery stage**		**Replication I stage**		**Replication II stage**	
	**Cases** **(*N* = 995)**	**Controls** **(*N* = 1,008)**	**Cases** **(*N* = 1,156)**	**Controls** **(*N* = 1,273)**	**Cases** **(*N* = 1,248)**	**Controls** **(*N* = 2,366)**
Age, years						
Mean (SD)	65.1 (14.5)	61.5 (9.5)	64.5 (12.2)	63.6 (11.2)	64.9 (12.5)	58.7 (12.6)
Median (Range)	63.0 (27.0–89.0)	62.0 (41.0–79.0)	64.0 (26.0–96.0)	65.0 (27.0–90.0)	66.0 (16.0–96.0)	60.0 (28.0–89.0)
Gender						
Male	799 (80.3)	1,008 (100)	930 (80.5)	816 (64.1)	1,010 (80.9)	1,501 (63.4)
Female	196 (19.7)		226 (19.5)	457 (35.9)	238 (19.1)	865 (36.6)
Smoking status						
Never	617 (62.0)	455 (45.1)	632 (54.7)	882 (69.3)	573 (45.9)	1,613 (68.2)
Ever	370 (37.2)	507 (50.3)	473 (40.9)	384 (30.2)	470 (37.7)	742 (31.4)
Unclear	8 (0.8)	46 (4.6)	51 (4.4)	7 (0.5)	205 (16.4)	11 (0.4)
Grade						
Low grade	373 (37.5)		302 (26.1)		491 (39.4)	
High grade	551 (55.4)		288 (24.9)		673 (53.9)	
Other	71 (7.1)		566 (49.0)		84 (6.7)	
Stage						
Non-muscle invasive	735 (73.9)		689 (59.6)		784 (62.8)	
Muscle invasive	245 (24.6)		251 (21.7)		418 (33.5)	
Other	15 (1.5)		216 (18.7)		46 (3.7)	

**Figure 2:**
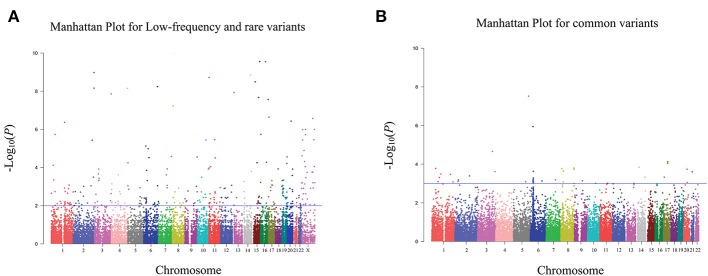
Manhattan plot for exome chip of bladder cancer in Han Chinese populations in the discovery stage. **(A)** Manhattan plot for low-frequency and rare variants. **(B)** Manhattan plot for common variants. The X axis represents the chromosomal position and the Y axis represents the –log_10_ *P*-value. The blue line indicates the filtering threshold of –log_10_ *P*-value. For low-frequency and rare variants, the blue line represented 0.01. For common variants, the blue line represented 0.001.

In the discovery stage, 995 bladder cancer cases and 1,008 controls were qualified for subsequent analysis. The principal component analyses revealed that cases and controls were genetically matched (Figure S2). And, as shown in the quantile-quantile plot (Figure S3), the inflation factor was 0.98. A representative cluster plot (rs35356162) generated by GenomeStudio was presented in Figure S4. Cluster plots for all SNVs in this study can be obtained from online available source data (https://doi.org/10.17632/bkvnsfgd4y.1). We determined the association of single variant with bladder cancer risk according to the following three categories: common variants, low-frequency and rare variants, and variants based on previously reported GWAS results. Manhattan plots for common variants, low-frequency, and rare variants are shown in Figure 2, with a line representing primary filtering threshold *P*-value. Different screening procedures were conducted between common and low-frequency and rare variants (Tables S4–S6). After filtering, we identified 4 common variants and 11 low-frequency and rare variants that were significantly associated with bladder cancer risk. In addition, we also genotyped additional 11 SNVs that were in previous reported GWA studies (21). Finally, 26 SNVs were selected for further validation in the additional cohorts. Details of these variants are presented in Table S8.

### Validation of Selected Variants in the Replication Stages

To evaluate the 26 SNVs selected in the discovery stage, they were genotyped in an independent replication I cohort of 1,156 cases and 1,273 controls using Sequenom MassArray Technology (Table 1). In this stage, only 3 SNVs achieved a *P* < 0.05, including a rare variant rs35356162 in the UHRF1 binding protein 1 gene (*UHRF1BP1*), and two previously reported variants: rs199701877 in the telomerase reverse transcriptase gene (*TERT*) and rs3736001 in the prostate stem cell antigen gene (*PSCA*). Genotyping details of these three SNVs are summarized in Table S9.

In the replication II stage, three SNVs, which survived in the replication I stage, were further validated in a cohort including 1,248 BCa cases and 2,366 cancer-free controls. Only *UHRF1BP1* rs35356162 and *PSCA* rs3736001 survived all three stages (Table S10). However, combined analysis after Bonferroni adjustment showed that only *UHRF1BP1* rs35356162 (OR = 4.332, 95% CI: 2.463 – 7.619, *P* = 3.62E-07 <7.93E-07) was identified as independent variant significantly associated with BCa risk in Han Chinese (Table 2).

**Table 2:** Summary of association with bladder cancer risk for rs35356162 and rs3736001 in the three-step stages and gene-based analysis of these two genes.

**SNV**	**Gene**	**Variant**	**Locus**	**Minor/Major allele**	**Stage**	Genotypes^*^		**MAF**		**OR (95% CI)**	***P*-Value**	***P-*Value**	**Number of SNVs in SKAT-O test**
						**Cases**	**Controls**	**Cases**	**Controls**			**(SKAT-O test)**	
rs35356162	*UHRF1BP1*	p. Gly152Val	6p21.31	T/G	Discovery	0/21/974	0/3/1,000	0.0106	0.0015	7.187 (2.137–24.172)	1.44E-03	4.47E-03	13
					Replication I	0/16/1,116	0/5/1,255	0.0071	0.0020	3.599 (1.314–9.855)	1.27E-02		
					Replication II	0/13/1,235	0/8/2,354	0.0052	0.0017	3.097 (1.280–7.493)	1.21E-02		
					**Combined**	**0/50/3,325**	**0/16/4609**	**0.0074**	**0.0017**	**4.332 (2.463–7.619)**	**3.62E-07**		
rs3736001	*PSCA*	p. Glu30Lys	8q24.3	A/G	Discovery	15/216/758	8/171/829	0.1244	0.0928	1.391 (1.137–1.703)	1.36E-03	1.30E-03	2
					Replication I	15/241/872	14/228/1,018	0.1201	0.1016	1.208 (1.007–1.448)	4.15E-02		
					Replication II	18/264/966	17/437/1,908	0.1202	0.0997	1.239 (1.060–1.447)	6.93E-03		
					**Combined**	**48/721/2,596**	**39/836/3755**	**0.1214**	**0.0987**	**1.265 (1.143–1.399)**	**5.00E-06**		

* Genotypes presented for rs35356162 in UHRF1BP1: TT/GT/GG and genotypes presented for rs3736001 in PSCA: AA/GA/GG.

Odds ratio and P-value derives from logistic regression in additive model.

*Bold values highlight the combined results of three stages*.

### Gene-Level Based Test

Considering the majority of individual variants were low-frequency or rare variants, and the limited power of single marker association analysis, we performed the SKAT-O test to evaluate the gene-level test as recommended (28). For the two variants passed the three-step filtering, the SKAT-O analysis based on exome chip genotyping demonstrated significant associations of *UHRF1BP1* (*P* = 4.47E-03) and *PSCA* (*P* = 1.30E-03) with BCa risk (Table 2). We also listed top 10 genes (with at least 10 SNVs in SKAT-O test) that were predicted to be highly associated with BCa risk in discovery stage, based on exome array results (Table S11).

### Population Genetics and Functional Prediction

**Figure 3:**
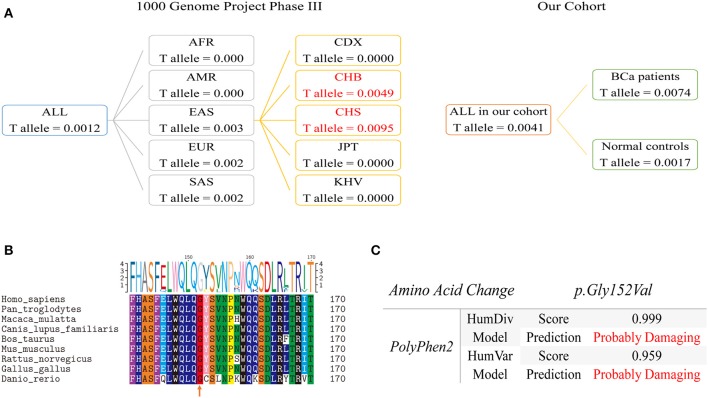
Population genetic comparison, evolutionary conservation analysis and functional prediction. **(A)** Left Panel: T allele frequency of rs35356162 in the 1000 Genomes Project. Frequencies of mainland Han Chinese subgroup are highlighted in red. Right Panel: the T allele frequency of rs35356162 in the present study, shown separately by bladder cancer cases and controls. **(B)** UHRF1BP1 p.Gly152 amino acid conservation analysis across species assessed with multiple sequences alignment. The orange arrow indicated the amino acid position which rs35356162 may alter. **(C)** *In silico* predicted effects of p.Gly152Val variant, assessed with PolyPhen2, by HumDiv and HumVar models. HumDiv model is a preferred model for evaluating rare alleles and dense mapping of regions identified by GWAS. HumVar model is a preferred model for diagnostics of Mendelian diseases which requires distinguishing mutations with drastic effects from all the remaining human variation, including abundant mildly deleterious alleles. In this study, HumDiv model is preferred, but we also presented result from HumVar model as a reference.

Population genetics of rs35356162 based on 1,000 Genomes Project Phase 3 showed that MAF of rs35356162 was 0.0012 globally, while a higher MAF (0.003) was observed in Eastern Asian population. The T allele frequencies of rs35356162 in Chinese Han Beijing population (0.0049) and Chinese Han south population (0.0097) were much higher than that detected in our study (Figure 3A).

Multiple sequences alignment across species revealed that p.Gly152 was a highly conserved amino acid site during evolution, which could lead to pathogenicity if altered (Figure 3B). The missense variant rs35356162 (p.Gly152Val) was predicted to be probably deleterious by PolyPhen2 (Figure 3C). Combined predictive results above indicated that this variant (p.Gly152Val) could probably change UHRF1BP1 protein function. Predictive results from other tools were shown in Figure S5.

### *In vitro* Functional Validation of *UHRF1BP1*

**Figure 4:**
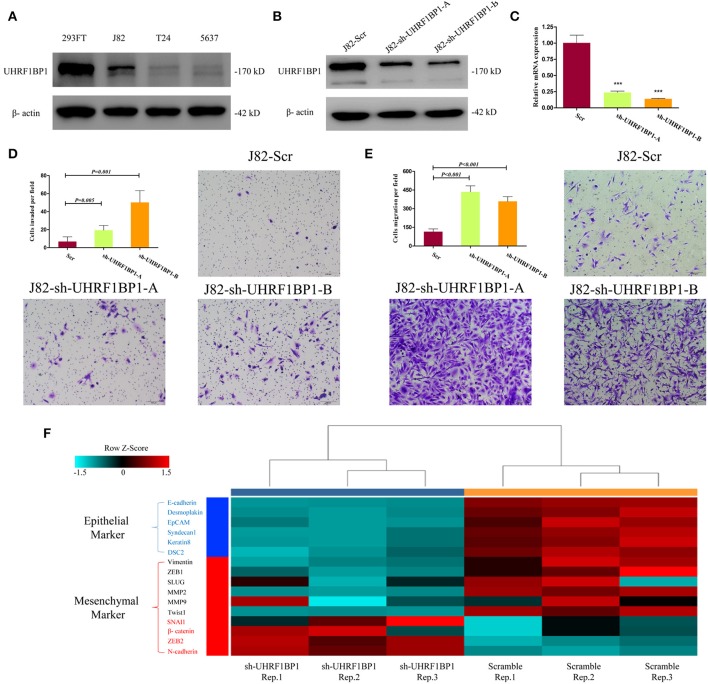
Down-regulation of UHRF1BP1 in J82 cell line can promote cell migration and invasion. **(A)** UHRF1BP1 is relatively highly expressed in J82 bladder cancer cell line, β-actin as a loading control. HEK293FT cell line acts as a positive control. **(B)** UHRF1BP1 protein expression is significantly inhibited by sh-UHRF1BP1-A&B. **(C)** UHRF1BP1 mRNA level is significantly inhibited by sh-UHRF1BP1-A&B. **(D)**. Representative images of invasion assay for J82 cells infected by scramble and sh-UHRF1BP1-A/B lentiviruses. **(E)** Representative images of migration assay for J82 cells infected by scramble and sh-UHRF1BP1-A/B lentiviruses. **(F)** Heatmap shows the mRNA expression differences of 16 genes involved in EMT between J82-Scr cells and J82-sh-UHRF1BP1-B cells in triplicates. This PCR array includes six epithelial markers (blue) and 10 mesenchymal markers (red). ^***^P < 0.001

Functional validation was performed to determine whether UHRF1BP1 played tumor-suppressive role in bladder cancer cell lines. Western blotting showed that J82 cell line was proficient in expressing UHRF1BP1 protein (Figure 4A). Two different short-hairpin RNA sequences both could remarkably down-regulate the expression of UHRF1BP1 in transcriptional level and translational level (Figures 4B,C). Down-regulation of UHRF1BP1 could sharply increase the ability of cell invasion and cell migration in both knockdown cell lines (Figures 4D,E). Quantitative real-time PCR analysis was performed to compare expression level of 16 epithelial-mesenchymal transition (EMT) related genes between scramble group and sh-UHRF1BP1-B group. The results further showed that all epithelial markers were down-regulated in the knockdown group, especially *E-cadherin, Desmoplakin*, and *EpCAM*, while mesenchymal markers *ZEB2* and *N-cadherin* were significantly up-regulated in the knockdown group (Figure 4F). Western blotting of representative EMT markers confirmed the results from PCR panels above (Figure S6). We also assessed proliferation ability alteration and cell-cycle distribution difference among scramble and knockdown groups, and achieved consistent results (Figure S7).

## Discussion

By examining multiple coding variants in a three-stage case–control study, we were the first to find that a low frequency variant in *UHRF1BP1*, rs35356162, increased bladder cancer susceptibility in Han Chinese population. The gene-level analysis indicated *UHRF1BP1* was strongly associated with BCa incidence, and functional experiments revealed a tumor suppressive function.

*UHRF1BP1* is located on chromosome 6p21 and encodes a protein with an unclear function. UHRF1BP1 was found to be an important part of the ICBP90 complex and a putative binding protein of UHRF1 in 2004 (29). Some studies demonstrated that several non-synonymous variants of *UHRF1BP1* were associated with systemic lupus erythematosus, both in European descendants and Chinese populations (30, 31). The role of UHRF1BP1 in cancer was initially investigated by a Japanese research group. They found that the interaction of UHRF1 with UHRF1BP1 may lead to relocation of UHRF1, while overexpression of UHRF1BP1 appeared to inhibit cell growth in colon cancer cell lines (29), which indicated that UHRF1BP1 could act as a tumor suppressor. Results in our study are consistent with previous deduction. Down-regulation of UHRF1BP1 expression in bladder cancer cell lines promoted invasion and migration, probably through EMT. Down-regulation of UHRF1BP1 expression can also promote cell proliferation, but perhaps not by regulating cell cycle. Hence, further studies should explore detailed mechanisms of tumor suppression ability, especially the interaction between UHRF1BP1 and UHRF1, which may play important roles in tumor DNA methylation transferring and other epigenetic events (32–36).

Population genetic comparison showed that the T allele frequency of rs35356161 in our study cohorts was lower than that in CHB or CHS cohort in the 1,000 Genomes Project. As described, the CHB population was collected in Beijing surrounding areas and the CHS population was recruited in Hunan and Fujian Provinces (37). However, participants of the present study were collected in Yangtze River Delta. The frequency difference may reflect heterogeneity of genetic background in Han Chinese population during the migration and fusion of nationalities in Chinese history, to some extent.

Previous GWAS studies reported that *PSCA* rs2294008 and rs2978974 conferred susceptibility to BCa in Caucasians and that the T allele of rs2294008 was associated with increased *PSCA* mRNA expression in both BCa tissues and normal bladder tissues (9, 38). Wang et al. validated the association between rs2294008 and increased BCa risk in a Han Chinese population (16). In our study, we observed that another *PSCA* variant, rs3736001, might also increase BCa risk, although this variant did not reach statistical significance. Given the obvious importance of *PSCA* polymorphisms in bladder cancer, functional studies of larger samples are warranted to delineate the precise effect of PSCA on bladder cancer.

Although the discovery stage had a relatively small sample size and limited statistical power, based on our calculation, we could also have a 0.98 power to detect the SNV with an OR of 4 and a frequency of 0.01 in control. A major strength of the present study is a large collection of Han Chinese case-control studies including a total of 3,399 bladder cancer patients and 4,647 controls in a three-stage study design, which ensures reliability of the results. However, there are certainly some limitations. Firstly, there was no female individual in the control group of discovery stage. This may lead to a selection bias. Secondly, apart from principal component analysis and clinical ethnic information collection, we did not perform other analyses to test genetic consistency in our study. This may reduce the reliability of the association study if without following multi-stage replication and functional analysis. Thirdly, we only performed basic functional evaluation of UHRF1BP1 in bladder cancer cell lines without mechanism exploration. Single amino acid-mutated plasmid and wild-type plasmid of UHRF1BP1 should have been constructed to do more precise investigation to further understand the role of UHRF1BP1 in cancer biological process. Finally, we did not collect detailed clinical and pathological data well, such as TNM staging, tumor multifocality and carcinoma *in situ*. We only focused on cancer risk correlation and missed the association between clinicopathological factors and SNVs, and also reduced the feasibility and reliability of subgroup analysis. Because of many missing data in clinical and pathological information, we only performed univariate logistic regression analysis to estimate the association between single variant and bladder cancer risk in Chinese population, without adjustment of clinicopathological features.

In conclusion, we found that a rare variant of *UHRF1BP1*, rs35356162, could increase bladder cancer risk in Han Chinese population. Our findings highlight the value of low-frequency and rare variants in identifying BCa associated genetic variation and cast a new light in BCa epidemiological screening and prevention.

## URL

R Statistical software: http://www.R-project.org/; Illumina: http://www.illumina.com/; Exome chip design: http://genome.sph.umich.edu/wiki/Exome_Chip_Design/;

PLINK: http://pngu.mgh.harvard.edu/~purcell/plink/; EIGENSOFT: http://genepath.med.harvard.edu/reich/Software.htm; PolyPhen2: http://genetics.bwh.harvard.edu/pph2/; CTex: http://www.ctex.org/HomePage/; ChinaPCa: http://www.chinapca.org.

## Data Availability Statement

The datasets generated for this study can be found in Mendeley Data, https://doi.org/10.17632/bkvnsfgd4y.1.

## Ethics Statement

This study was carried out in accordance with the recommendations of the Scientific and Ethical Committee of Fudan University Shanghai Cancer Center with written informed consent from all subjects. All subjects gave written informed consent in accordance with the Declaration of Helsinki. The protocol was approved by the Scientific and Ethical Committee of Fudan University Shanghai Cancer Center.

## Author Contributions

DY and YZ conceived and designed the research. JW, MW, and JX acquired the data. JW drafted the manuscript. JW and GZ performed experiments. HC and CG analyzed the data. QD and QW contributed reagents, materials, and analysis tools.

### Conflict of Interest

The authors declare that the research was conducted in the absence of any commercial or financial relationships that could be construed as a potential conflict of interest.
