# Supplementary material for: The Rare Variant rs35356162 in UHRF1BP1 Increases Bladder Cancer Risk in Han Chinese Population
Source: Front Oncol. 2020 Feb 11;10:134. doi: 10.3389/fonc.2020.00134 (PMC7026461; doi:10.3389/fonc.2020.00134)
Supplement: Supplementary file 1 [file Data_Sheet_1.pdf]

Supplementary Files

The rare variant rs35356162 in *UHRF1BP1* increases bladder cancer risk in Han Chinese population

Junlong Wu [1,2,#], Meilin Wang [3,#], Haitao Chen [4,5,#], Jianfeng Xu [5,6,#], Guiming Zhang [7], Chengyuan Gu [1,2], Qiang Ding [5], Qingyi Wei [8,9], Yao Zhu [1,2,*], Dingwei Ye [1,2,*]

# These authors contributed equally to this work.

* Corresponding authors

1. Department of Urology, Fudan University Shanghai Cancer Center, Shanghai, 200032, China

2. Department of Oncology, Shanghai Medical College, Fudan University, Shanghai, China

3. Department of Genetic Toxicology, the key laboratory of Modern Toxicology of Ministry of Education, School of Public Health, Nanjing Medical University, Nanjing, China

4. State Key Laboratory of Organ Failure Research, Guangdong Key Laboratory of Viral Hepatitis Research, Department of Infectious Diseases and Hepatology Unit, Nanfang Hospital, Southern Medical University, GuangZhou, China.

5. Fudan Institute of Urology, Huashan Hospital, Fudan University, Shanghai, China

6. Center for Cancer Genomics, Wake Forest School of Medicine, Winston-Salem, NC, USA

7. Department of Urology, the Affiliated Hospital of Qingdao University, Qingdao, China

8. Cancer Institute, Fudan University Shanghai Cancer Center, Shanghai, China.

9. Duke Cancer Institute, Duke University Medical Center, Durham, NC, USA.

*Address for Correspondence:
Prof. Ding-Wei Ye, M.D. (dingwei_ye1963@163.com) and Dr. Yao Zhu M.D. (mailzhuyao@163.com), Department of Urology, Fudan University Shanghai Cancer Center, No. 270 Dong'an Road, Shanghai 200032, People's Republic of China. Tel: 86-21-64175590-2807; Fax: 86-21-64434556.

Supporting Information List

Supplementary Materials and methods

Supplementary Figures

S1 Fig. Identity-by-state similarity plot. The X axis represents pairs of individuals, while the Y axis shows similarity score. Participants with a similarity score over 0.99 are excluded in the discovery stage.

S2 Fig. Principal component analysis (PCA) representative plot in the discovery stage. The first two eigen vectors of PCA were depicted, which indicates that cases and controls were genetically matched.

S3 Fig. Quantile-quantile plot. The quantile-quantile (Q-Q) plot of the expected and observed *P* values, and the shaded band represents 95% CI. The deviation of the QQ plot may be caused by the population stratification or the extremely significant signals in certain chromosomes, like 6p21. The inflation factor of our population is 0.98, indicating that the study population is a relatively genetically matched population.

S4 Fig. A representative cluster plot of rs35356162. Left panel: Cartesian Coordinates Cluster plot of rs35356162; Right Panel: Polar Coordinates Cluster plot of rs35356162.

S5 Fig. In silico predicted effects of p.Gly152Val variant, assessed with SIFT, PROVEAN, CADD and MutationTaster.

S6 Fig. Western blotting of representative markers of EMT in knockdown and wild-type UHRF1BP1 bladder cancer cells. Epithelial markers E-cadherin and EpCAM are down-regulated in UHRF1BP1 knockdown cells. Mesenchymal marker N-cadherin is up-regulated in UHRF1BP2 knockdown cell.

S7 Fig. Knockdown of UHRF1BP1 in J82 cells can promote cell proliferation. (A) CCK-8 analysis shows significant increased proliferation ability in J82 sh-UHRF1BP1-A&B cells than J82 scramble cells. (B) Cell cycle distribution of J82 cells upon knockdown of UHRF1BP1 expression. (C) Representative images of cell

cycle distribution in J82 scramble and J82 sh-UHRF1BP1-A&B cells.

Supplementary Tables

S1 Table. Association of previously identified SNVs for bladder cancer risk.

S2 Table. Sample Quality Control

S3 Table. SNVs Quality Control

S4 Table. Filtering of Common variants

S5 Table. Filtering of Low-frequency and rare variants

S6 Table. Filtering of reported variants in our previous GWAS data

S7 Table. Genes and corresponding qRT-PCR primers used in this study.

S8 Table. Variants associated with bladder cancer risk identified in the discovery stage.

S9 Table. Variants associated with bladder cancer risk identified in the replication I stage.

S10 Table. Variants associated with bladder cancer risk identified in the replication II stage.

S11 Table. Top 10 genes (with at least 10 SNVs in SKAT-O test) associated with bladder cancer identified by the SKAT-O test in the discovery stage.

S12 Table. Important genetic alteration backgrounds of bladder cancer cell lines used in this study

Supplementary Materials and methods

Recruitment criteria of bladder cancer cases and normal controls

The discovery stage included 1,019 bladder cancer cases recruited from Fudan University Shanghai Cancer Center (FUSCC) between January 2007 and May 2013, and 1,008 controls recruited from four regions of Shanghai from April 2010 who were shared in the Chinese Consortium for Prostate Cancer Genetics. In replication I stage, 1,156 bladder cancer cases and 1,273 controls were included who mainly derived from Nanjing and surrounding areas. The replication II stage included 1,248 cases and 2,366 controls recruited from four hospitals in Shanghai including FUSCC, Huashan Hospital, Changhai Hospital, and Zhongshan Hospital.

Individuals in control group were genetically unrelated to BCa patients. They also had no history of any cancer or any sign that indicates bladder cancer, including hematuria, bladder occupying lesions or bladder wall thickening detected by imaging examinations previously.

Study design supplements

The 3-stage association study design is similar to our previously published work [1]. In this work, due to the limited cohort in discovery stage, we did not aim to conclude Bonferroni corrected significant variants in only one stage. Filtering P values in all these 3 stages are self-defined broad cutoff, aiming to include more suggestive associated variants and potentially to be proved significant in following stage(s). This three-phase study is like a funnel-shaped step-by-step screening study, with the goal of finding new significant association variants. So, we used Bonferroni cutoff calculated by 0.05/63,047 SNVs (7.93E-07), for three stages combined to screen out significant variant.

Procedures of quality control for samples and SNVs in discovery stage

(1) Removing duplicate samples and samples who showed familial relationships according to identity-by-state (IBS) analysis (IBS similarity score > 0.99, performed

using PLINK v1.07 IBS similarity matrix).

(2) Removing samples without complete clinical information.

(3) Removing SNVs that had a genotyping rate less than 99% in total cohort, in cases or controls separately.

(4) Removing SNVs that had a P value less than 1.0E-03 of Hardy-Weinberg Equilibrium (HWE) test in controls.

(5) Detailed filtering procedures of SNVs described in **S4-6 Tables** could be referred to previous published work [1].

Cell lines and culture

The human bladder cancer cell lines J82, 5637, T24 and embryonic kidney cell line HEK293FT were obtained from the Cell Bank of Shanghai Institutes of Biological Sciences, Chinese Academy of Sciences. These cell line were all characterized by Genetic Testing Biotechnology Corporation (Suzhou, China) using short tandem repeat (STR) markers in June 2017. Important genetic alteration backgrounds (including mutations and gene fusions) of these bladder urothelial carcinoma cell lines were obtained from cBioPortal Platform (https://www.cbioportal.org/) based on Cancer Cell Line Encyclopedia data and were shown in **S12 Table**. J82, 5637, T24 and HEK293FT were cultured in Minimum Essential Medium (MEM), RPMI 1640 medium, McCoy's 5A medium and DMEM medium respectively, as ATCC suggested, with 10% fetal bovine serum (S181P, Biowest LCC) at 37℃ in an atmosphere of 5% CO2.

Plasmids constructs and lentivirus preparation

Lentiviral vector pLKO.1 TRC (Addgene 10879) was used for constructing recombinant lentiviruses to knockdown certain gene. Two different oligonucleotides encoding hairpin precursors for sh-UHRF1BP1-A (5'-GCCTCGTAGATTCAGAGCTAT-3') and sh-UHRF1BP1-B (5'-GCAGGTGATAGCTGCAAACAT -3') were cloned. A scrambled sequence (Scr) was used as a control which did not affect transcriptome in cell lines after been

transfected.

We used helper plasmids, pSPAX2 (Addgene plasmid 12260) and pMD2.G (Addgene plasmid 12259) to co-transfected with pLKO.1-based plasmids into HEK293FT cells to package recombinant lentiviruses, using Lipofectamin 2000 (Invitrogen, Carlsbad, CA, USA) according to manufacturers' protocols. Supernatants from co-transfections were used for lentiviral infection of cultured bladder cancer cell lines.

RNA extraction, reverse transcription and quantitative real-time PCR analysis

Total RNA of different cell lines was extracted using TRIzol reagent (15596-026, Invitrogen). A PrimeScript RT reagent kit (K1622, Thermo Scientific) was used to synthesize first-strand cDNA from total RNA. After that, we performed SYBR Green real-time PCR analysis using ABI 7900HT machine (Applied Biosystems, Foster City, California, USA). We used β-actin mRNA expression level as a reference. Target genes' mRNA expression level was normalized to that of β-actin. The primers for qRT-PCR analysis were synthesized by Sangon (Shanghai, P.R. China), sequences of which are shown in **S7 Table**. Heatmap of relative mRNA expression of 16 genes was plotted using Heatmap.2 function in R "gplot" package.

Antibodies for western blot

Antibodies used in western blot included anti-UHRF1BP1 (ab199849, Abcam, Cambridge, UK), anti-β-actin (ab8227, Abcam, Cambridge, UK), anti-E-cadherin (#14472, Cell Signaling Technology, Danvers, MA), anti-N-cadherin (#13116, Cell Signaling Technology, Danvers, MA) and anti-EpCAM (#93790, Cell Signaling Technology, Danvers, MA).

Migration and invasion assay

We cultured pre-treated J82 cells in a 6-well plate until they reached an over 90% density. Then, the cells were trypsinized and resuspended in the MEM medium containing 0.5% FBS at 1x105 cells/ml, and 200 ul of cell suspension was seeded

into Tranwell inserts (8 μm, corning Costar) with or without Matrigel coating (BD Bioscience) to perform in vitro invasion or migration assay, respectively.
We added 500 μl MEM medium supplemented with 10% FBS in the lower chamber. Cells on the upper surface were scrapped off with a cotton swab after culturing for 36 hours. Then, the membranes were fixed and stained with Wtight-Giemsa staining for 15 minutes. Invasive or migrated cells were quantified by counting the number of cells which penetrated the membrane.

Cell proliferation assay

Cell proliferation was measured using a CCK-8 kit (Dojindo, Kumamoto, Japan). J82 cells infected with lentiveruses were seeded into 96-well plates in 100 μl of MEM medium with 10% FBS in triplicates, and were incubated at 37℃ in 5% CO2. After 24, 48, 72 and 96 hours, the medium was replaced with 90 μl complete MEM medium and 10 μl CCK-8 solution. Then, cells were incubated for 2 hours at 37℃ in 5% CO2 and absorbance at 450 nm was measured.

Cell cycle analysis

Cell cycle distribution was measured by fluorescence-activated cell sorting (FACS) analysis. Infected cells were seeded into a 6 well plate and after reaching a density 70%, cells were fixed with -20℃ cooled 70% ethanol overnight at 4℃ for subsequent cell cycle analysis. Cells were washed with PBS and treated with Ribonuclease A (100ug/ml, D202, Dalian, China) at 37℃ for 30 min and stained with propidium iodide (PI, 50ug/ml) at room temperature for 15 min. PI-stained cells were run on FC500-MPPL Beckman Coulter and cell cycle data were analyzed using MultiCycle AV DNA Analysis software.

S1 Fig

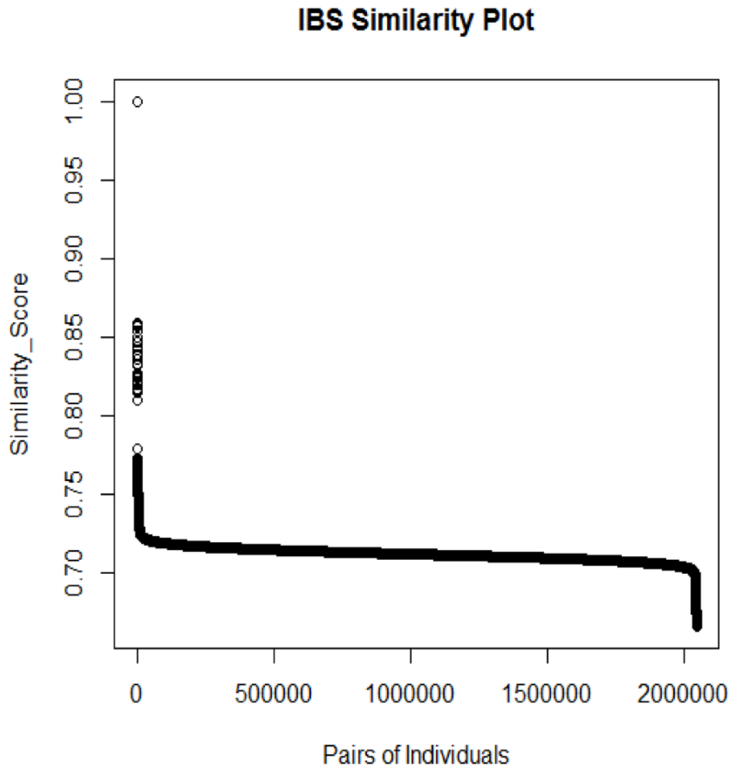
IBS Similarity Plot
Similarity_Score
1.00
0.95
0.90
0.85
0.80
0.75
0.70
0
500000
1000000
1500000
2000000
Pairs of Individuals

S2 Fig

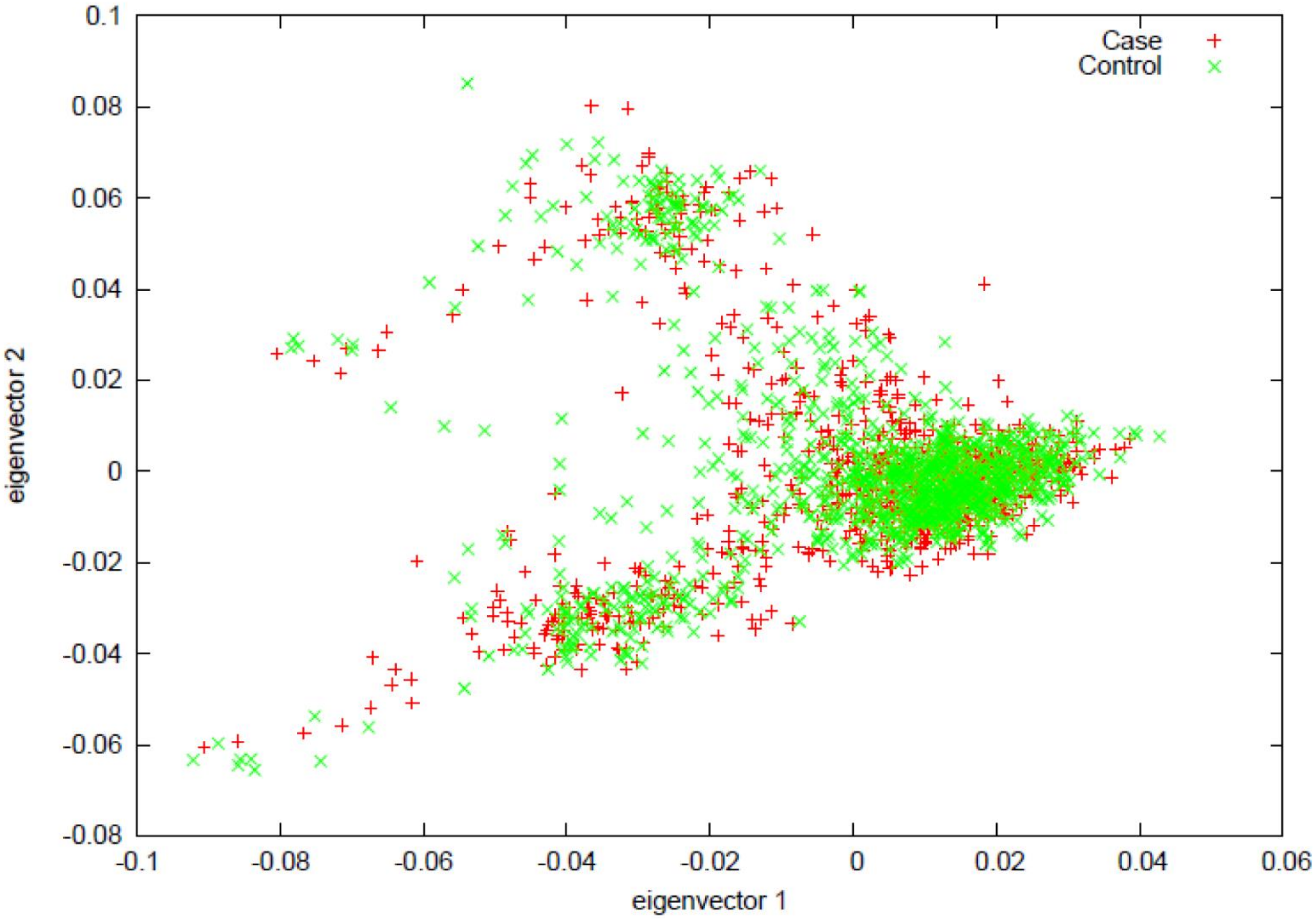

Case
Control
0.1
0.08
0.06
0.04
0.02
0
-0.02
-0.04
-0.06
-0.08
eigenvector 2
-0.1
-0.08
-0.06
-0.04
-0.02
0
0.02
0.04
0.06
eigenvector 1

S3 Fig

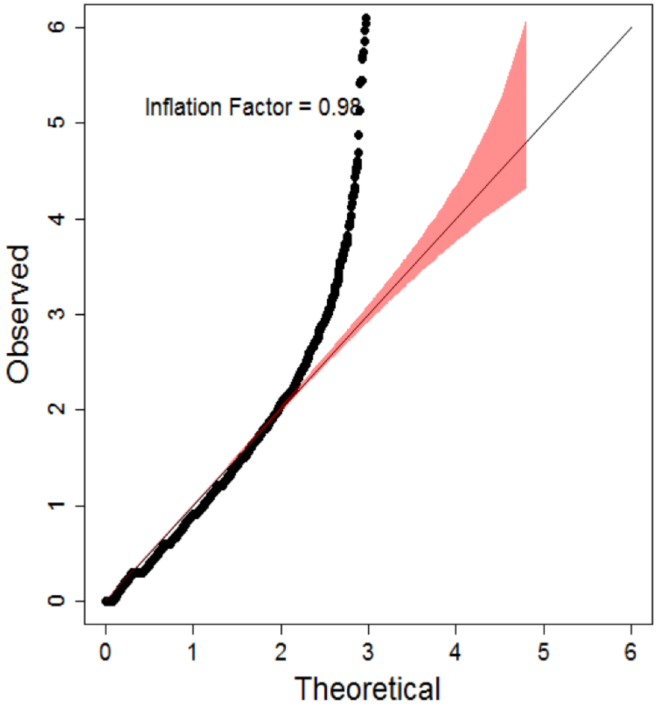

S4 Fig

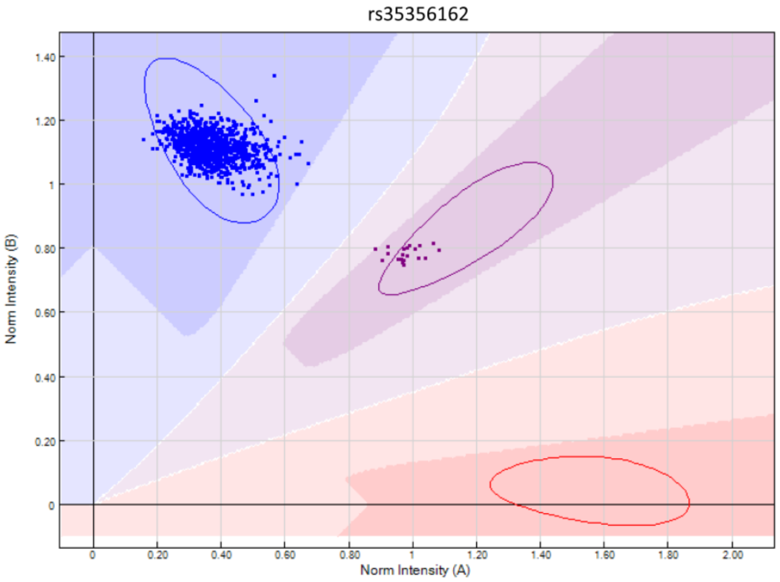

Cartesian Coordinates Cluster Plot

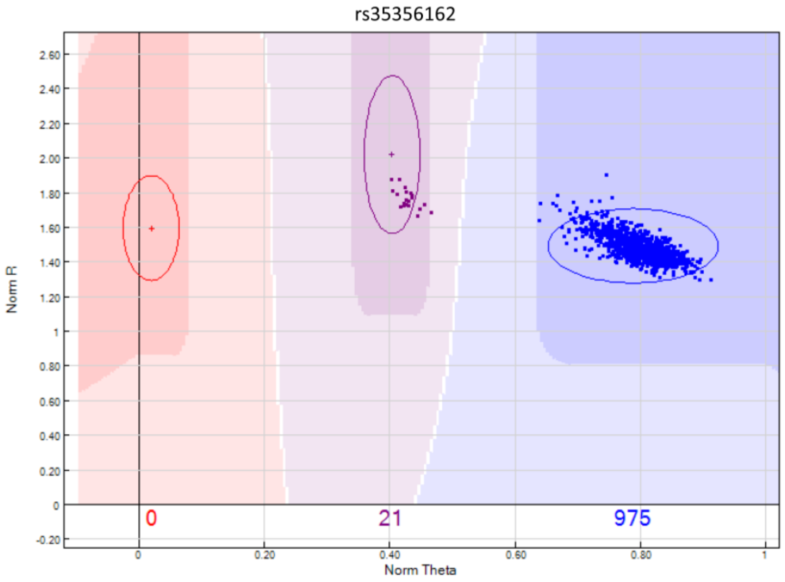

Polar Coordinates Cluster Plot

S5 Fig.

Amino Acid Change		*p. Gly152Val*
SIFT	Score	0.949
	Prediction	Tolerated
	Basis	Evolutionary conservation
PROVEAN	Score	0.68
	Prediction	Neutral
	Basis	Alignment and measurement of similarity between variant sequence and protein sequence homolog
CADD	Score	23.9
	Prediction	deleteriousness
	Basis	Contrasts annotations of fixed/nearly fixed derived alleles in humans with simulated variants
MUTATIONTASTER	Score	--
	Prediction	disease causing
	Basis	Protein structure/function and evolutionary conservation

S6 Fig

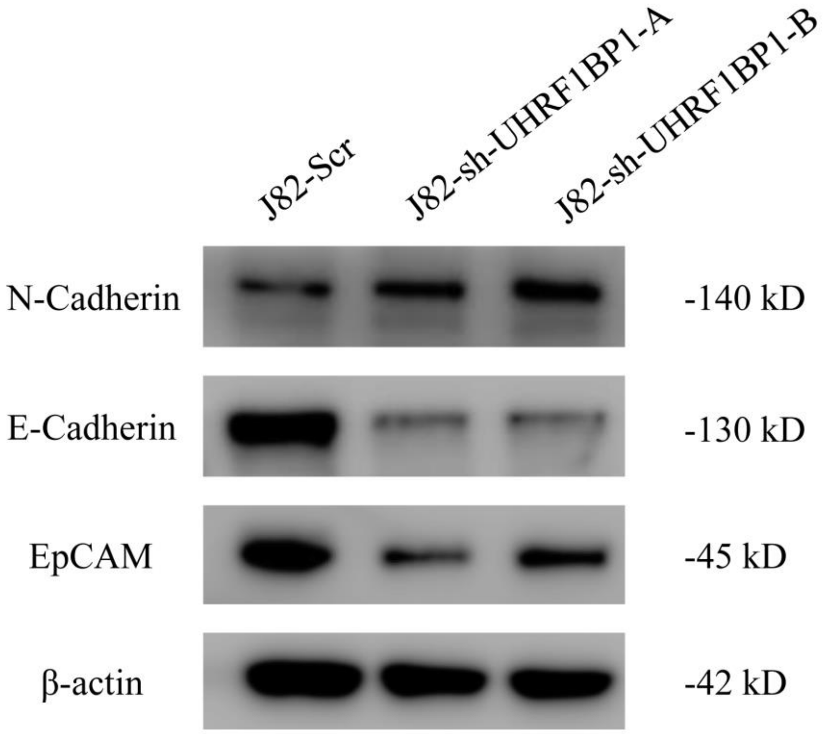
J82-Scr
J82-sh-UHRF1BP1-A
J82-sh-UHRF1BP1-B
N-Cadherin
E-Cadherin
EpCAM
β-actin
-140 kD
-130 kD
-45 kD
-42 kD

S7 Fig

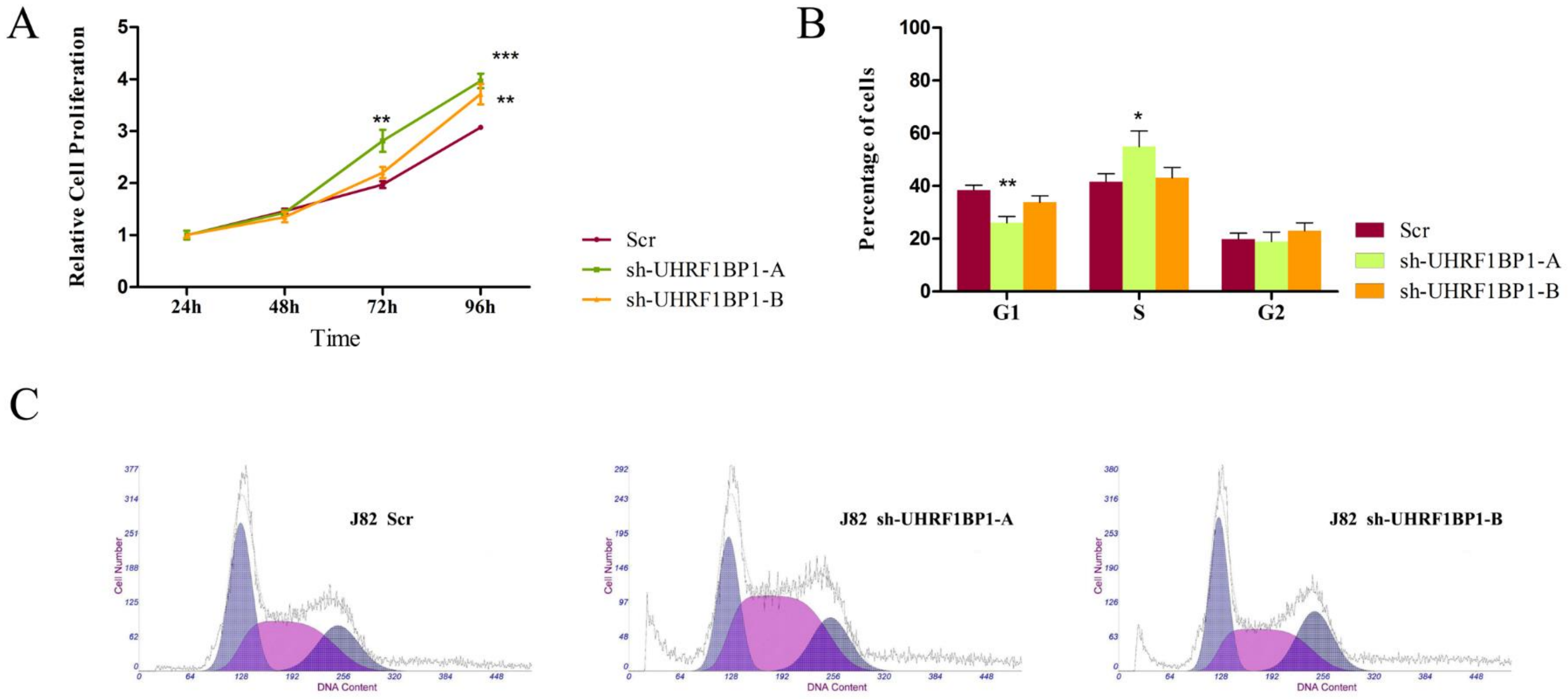
A
Relative Cell Proliferation
Time
24h
48h
72h
96h
Scr
sh-UHRF1BP1-A
sh-UHRF1BP1-B
B
Percentage of cells
G1
S
G2
Scr
sh-UHRF1BP1-A
sh-UHRF1BP1-B
C
J82 Scr
J82 sh-UHRF1BP1-A
J82 sh-UHRF1BP1-B
Cell Number
DNA Content

S1 Table. Association of previously identified SNPs for bladder cancer risk.

SNV	Locus	Gene	Previous reports					Discovery stage in this study				
			Population	Allele [a]	Risk allele	RAF [b]	OR	Allele	Risk allele	RAF	OR*	P value*
rs9642880	8q24.21	MYC	European	G/T	T	0.450	1.21	G/T	T	0.297	1.16	0.028
rs710521	3q28	TP63	European	A/G	A	0.730	1.19	A/G	A	0.788	1.10	0.236
rs2736098	5p15.33	TERT	European	G/A	A	0.255	1.08	--	--	--	--	--
rs401681	5p15.33	CLPTM1L	European	C/T	C	0.535	1.12	C/T	C	0.664	1.23	0.003
rs2294008	8q24.3	PSCA	European	C/T	T	0.463	1.15	C/T	T	0.252	1.27	0.001
rs798766	4p16.3	TACC3, FGFR3	European	C/T	T	0.190	1.24	C/T	T	0.111	1.28	0.010
rs1014971	22q13.1	CBX6, APOBEC3A	European	T/C	T	0.620	1.14	--	--	--	--	--
rs8102137	19q12	CCNE1	European	T/C	C	0.330	1.33	T/C	C	0.091	1.28	0.018
rs11892031	2q37.1	UGT1A	European	A/C	A	0.920	1.19	A/C	A	0.963	1.96	0.001
rs1495741	8p22	NAT2	European	A/G	A	0.800	1.15	G/A	A	0.404	1.23	0.001
--	1p13.3	GSTM1	European	Null/non-null	Null	0.510	1.46	--	--	--	--	--
rs17674580	18q12.3	SLC14A1	European	C/T	T	0.430	1.16	C/T	T	0.072	1.38	0.005
rs10936599	3q26.2	MYNN	European	C/T	C	0.760	1.18	T/C	C	0.432	1.21	0.003
rs907611	11p15.5	LSP1	European	G/A	A	0.320	1.15	G/A	A	0.240	1.15	0.059
rs62185668	20p12.2	JAG1	European	C/A	A	0.236	1.19	--	--	--	--	--
rs11543198	15q24	CYP1A2	European	G/A	G	0.778	1.41	--	--	--	--	--
rs4907479	13q34	MCF2L	European	G/A	A	0.261	1.11	--	--	--	--	--

*Odds ratio and P value derives from logistic regression in additive model.

a. Major/minor allele

b. Risk allele frequency in the controls

S2 Table. Sample Quality Control

	Discovery stage	
	Cases	Controls
Pre-QC	1019	1008
Relatives (IBS>0.99)	20	0
Duplicates	3	0
Genotype call rate < 0.99	0	0
Incomplete clinical data	1	0
Total exclusions	24	0
Post-QC	995	1008

Note: We used PLINK v1.07 to investigate duplicates and genetic relatedness by performing identity-by-state (IBS) analysis. Genotype call rate was generated automatically by standard Illumina's GenTrain version 2.0 clustering algorithm using GenomeStudio software (V2011.1)

S3 Table. SNVs Quality Control

QC	Number of SNPs left
Pre-QC	270241
Combined genotyping missing rate >0.02	29050
Cases genotyping missing rate >0.02	138
Controls genotyping missing rate >0.02	438
None genotyping polymorphism	176887
Control HWE *P* <0.001	636
Total exclusions	207194
Post-QC	63047

Note: Firstly, we excluded SNVs which had a genotyping missing rate > 0.02 in 995 cases and/or 1008 controls. Then we excluded SNVs which were not detected polymorphism (different allele) in cases and controls. Finally, we excluded SNVs deviated from Hardy–Weinberg equilibrium (HWE) in the controls at P < 0.001.

S4 Table. Filtering of Common variants

	Filtering method	Left SNPs
Step 1		
1	MAF > 0.05 in cases and controls	
2	*P* < 0.001	
		46 SNPs
Step 2		
3	MAF in controls vs. MAF in previous GWAS Chinese studies ~0.02 [a]	
4	In the same region or gene [b]	
		20 SNPs
Step 3		
5	GWAS consistant [c]	
6	*P* < 1.0E-08 in GWAS [d]	
		4 SNPs

a. Compare the MAF between groups. The allele frequencies of the selected SNVs in the controls were compared with the results of other published GWAS conducted for Chinese populations [2 3], and SNVs were excluded if the differences >0.02 in these controls [1].

b. If there are several SNVs left in the same region or gene, we choose one of them to enter the next filtering step based on P value.

c. If a certain SNV is in the gene or region which has been reported to be associated with bladder cancer risk previously, the SNV will be discarded.

d. If a certain SNV has been reported to reach a P value less than 1.0E-08 in previous GWAS, the SNV will be discarded.

S5 Table. Filtering of Low-frequency and rare variants

	Filtering method	Left SNPs
Step 1		
1	MAF ≤ 0.05 in cases and controls	
2	$P < 0.01$	
3	MAF in cases > MAF in controls	
		227 SNPs
Step 2		
4	MAF in controls vs. MAF in previous GWAS Chinese studies ~0.02 [a]	
5	In the same region or gene [b]	
		137 SNPs
Step 3		
7	Removing variants in Chr 23	
		105 SNPs
Step 4		
8	Clumping analyses [d]	
		11 SNPs

a. Compare the MAF between groups. The allele frequencies of the selected SNVs in the controls were compared with the results of other published GWAS conducted for Chinese populations [2 3], and SNVs were excluded if the differences >0.02 in these controls [1].

b. If there are several SNVs left in the same region or gene, we choose one of them to enter the next filtering step based on P value.

c. Using PLINK LD-based result clumping procedure (default) to perform clustering (r^2=0.2). See URL.

S6 Table. Filtering of reported variants in our previous GWAS data

	Filtering method	Left SNPs
1	*P* < 0.05 in discovery stage	
		11 SNPs

S7 Table. Genes and corresponding qRT-PCR primers used in this study.

Gene Symbol	Forward qRT-PCR primer	Reverse qRT-PCR primer
β-actin	CATGTACGTTGCTATCCAGGC	CTCCTTAATGTCACGCACGAT
UHRF1BP1	TGTGTCTGGATAAGGTAGAGGTG	GGCAAAGCCATATTCACTCTGT
E-cadherin	ATTTTTCCCTCGACACCCGAT	TCCCAGGCGTAGACCAAGA
EpCAM	TGATCCTGACTGCGATGAGAG	CTTGTCTGTTCTTCTGACCCC
Desmoplakin	TCGTGCAGCCTGAATTGAAGT	CCTGGGCAAAACACTCATCC
Keratin 8	CAGAAGTCCTACAAGGTGTCCA	CTCTGGTTGACCGTAACTGCG
Syndecan 1	ACGGCTATTCCCACGTCTC	TCTGGCAGGACTACAGCCTC
DSC2	ACACGGCCCAAAACTATACCA	TTTCCAGTGTCTCTCTCCACATA
N-cadherin	TGCGGTACAGTGTAACTGGG	GAAACCGGGCTATCTGCTCG
β-catenin	AGCTTCCAGACACGCTATCAT	CGGTACAACGAGCTGTTTCTAC
Zeb 1	CAGCTTGATACCTGTGAATGGG	TATCTGTGGTCGTGTGGGACT
Zeb 2	CAAGAGGCGCAAACAAGCC	GGTTGGCAATACCGTCATCC
Slug (SNAI2)	CGAACTGGACACACATACAGTG	CTGAGGATCTCTGGTTGTGGT
Snail(SNAI1)	TCGGAAGCCTAACTACAGCGA	AGATGAGCATTGGCAGCGAG
Twist1	GTCCGCAGTCTTACGAGGAG	GCTTGAGGGTCTGAATCTTGCT
MMP2	GATACCCCTTTGACGGTAAGGA	CCTTCTCCCAAGGTCCATAGC
MMP9	GGGACGCAGACATCGTCATC	TCGTCATCGTCGAAATGGGC
Vimentin	AGTCCACTGAGTACCGGAGAC	CATTTCACGCATCTGGCGTTC

S8 Table. Variants associated with bladder cancer risk identified in the discovery stage.

SNP	Gene	Chromosome	Major/Minor Allele	Combined MAF	Case MAF	Control MAF	OR	*P* Value	P^{HWE}
Common Variants									
rs1801265	*DPYD*	1	G/A	0.0755	0.0906	0.0605	1.528	4.49E-04	0.864
rs2303077	*HMMR*	5	C/G	0.0852	0.1101	0.0610	1.963	2.27E-08	0.335
rs111303879	*OR2J2*	6	A/G	0.3885	0.3508	0.4259	0.616	1.34E-09	0.460
rs7144658	*FOXA1*	14	C/T	0.1328	0.1533	0.1126	1.432	1.43E-04	0.945
Low-frequency and rare variants									
bs1_186094772	*HMCN1*	1	C/T	0.0042	0.0070	0.0015	4.722	1.50E-02	0.962
rs116818533	*BIRC6*	2	A/T	0.0018	0.0035	0	NA	7.43E-03*	-
rs114453306	*CTDSPL*	3	C/T	0.0028	0.0050	0.0005	10.520	2.51E-02	0.987
bs6_31838588	*SLC44A4*	6	C/T	0.0110	0.0222	0	NA	3.20E-14*	-
rs35356162	*UHRF1BP1*	6	G/T	0.0060	0.0106	0.0015	7.187	1.44E-03	0.962
bs12_126068523	*TMEM132B*	12	G/A	0.0035	0.0066	0.0005	8.269	3.68E-02	0.987
bs14_20585958	*OR4K17*	14	G/T	0.0023	0.0045	0	NA	1.83E-03*	-
bs14_24617068	*RNF31*	14	C/T	0.0110	0.0222	0	NA	2.99E-14*	-
rs78482832	*IL16*	15	A/G	0.0063	0.0127	0	NA	2.12E-08*	-
rs80158709	*MRPL28*	16	C/T	0.0070	0.0106	0.0035	3.138	9.30E-03	0.912
bs17_26918889	*SPAG5*	17	G/A	0.0162	0.0226	0.0099	2.335	1.90E-03	0.750
Variants based on previously reported GWAS results									
rs78991672	*SLC6A18*	5	A/G	0.0360	0.0261	0.0456	0.552	8.07E-04	0.943
rs117052364	*SLC6A18*	5	G/A	0.0190	0.0146	0.0233	0.629	4.97E-02	0.532
rs4073918	*SLC6A18*	5	C/T	0.2800	0.2636	0.2961	0.846	1.91E-02	0.717
bs5_1294163	*TERT*	5	C/T	0.0035	0.0070	0	NA	5.64E-05*	-

rs10100935	*TSNARE1*	8	T/C	0.3917	0.3759	0.4072	0.875	4.13E-02	0.349
rs3736001	*PSCA*	8	G/A	0.1084	0.1244	0.0928	1.391	1.36E-03	0.801
bs4_1657232	*FAM53A*	4	G/A	0.0020	0.0035	0.0005	6.808	3.77E-02	0.987
bs19_30165063	*PLEKHF1*	19	A/G	0.0072	0.0040	0.0104	0.382	2.15E-02	0.738
rs78122518	*USP40*	2	C/T	0.0764	0.0885	0.0645	1.394	5.36E-03	0.345
rs79471829	*USP40*	2	A/T	0.0765	0.0886	0.0645	1.396	5.20E-03	0.345
rs1799930	*NAT2*	8	G/A	0.2258	0.2460	0.2059	1.255	2.78E-03	0.741

*: Fisher exact test, because of no polymorphism detected in control group.

Odds ratio and *P* value derives from logistic regression in additive model.

P^{HWE}: *P* value derives from Hardy-Weinberg Equilibrium test.

S9 Table. Variants associated with bladder cancer risk identified in the replication I stage.

SNP	Gene	Chromosome	Minor/Major Allele	Combined MAF	Case MAF	Control MAF	OR	*P* Value
Low-frequency and rare variants								
rs35356162	*UHRF1BP1*	6	G/T	0.0044	0.0071	0.0020	3.599	1.27E-02
Variants based on previously reported GWAS results								
bs5_1294163	*TERT*	5	C/T	0.0025	0.0044	0.0008	4.727	1.76E-02
rs3736001	*PSCA*	8	C/T	0.1103	0.1201	0.1016	1.208	4.15E-02

Odds ratio and *P* value derives from logistic regression in additive model.

S10 Table. Variants associated with bladder cancer risk identified in the replication II stage.

SNP	Gene	Chromosome	Minor/Major Allele	Combined MAF	Case MAF	Control MAF	OR	*P* Value
Low-frequency and rare variants								
rs35356162	*UHRF1BP1*	6	G/T	0.0029	0.0052	0.0017	3.097	1.21E-02
Variants based on previously reported GWAS results								
rs3736001	*PSCA*	8	C/T	0.1068	0.1202	0.0997	1.239	6.93E-03

Odds ratio and *P* value derives from logistic regression in additive model.

S11 Table. Top 10 genes (with at least 10 SNVs in SKAT-O test) associated with bladder cancer identified by the SKAT-O test in the discovery stage.

Gene	Locus	Number of Markers (ALL)	Number of Markers (Tested)	P Value (SKAT-O test)	Rank $_{SKAT\text{-}O}$
RP1L1	8p23.1	26	26	3.23E-26	1
HLA-DQA2	6p21.32	35	35	9.22E-18	2
LTBP2	14q24.3	18	18	3.12E-13	3
SLC44A4	6p21.33	14	14	1.60E-11	4
ADAMTS14	10q22.1	12	12	3.09E-11	5
MAPKBP1	15q15.1	10	10	6.87E-11	6
KIF17	1p36.12	12	12	1.23E-10	7
CYP1A1	15q24.1	13	13	1.46E-10	8
MUC22	6p21.33	43	43	3.94E-10	9
CUX1	7q22.1	10	10	9.74E-10	10

S12 Table. Important genetic alteration backgrounds of bladder cancer cell lines used in this study*.

Cell Line	TP53	KDM6A	RB1	FGFR3
5637	R280T	no alteration	Y325*	no alteration
J82	E271K, K320N	C1361Y	X703_splice	K650E
T24	no alteration	no alteration	no alteration	no alteration

*Obtained from cBioPortal Platform (https://www.cbioportal.org/) based on Cancer Cell Line Encyclopedia data

Reference

1. Wang M, Li Z, Chu H, Lv Q, Ye D, Ding Q, Xu C, Guo J, Du M, Chen J, Song Z, Yin C, Qin C, Gu C, Zhu Y, Xia G, Liu F, Zhang Z, Yuan L, Fu G, Hu Z, Tong N, Shen J, Liu K, Sun J, Sun Y, Li J, Li X, Shen H, Xu J, Shi Y, Zhang Z. Genome-Wide Association Study of Bladder Cancer in a Chinese Cohort Reveals a New Susceptibility Locus at 5q12.3. Cancer research 2016;**76**(11):3277-84 doi: 10.1158/0008-5472.can-15-2564[published Online First: Epub Date]|.
2. Jiang DK, Sun J, Cao G, Liu Y, Lin D, Gao YZ, Ren WH, Long XD, Zhang H, Ma XP, Wang Z, Jiang W, Chen TY, Gao Y, Sun LD, Long JR, Huang HX, Wang D, Yu H, Zhang P, Tang LS, Peng B, Cai H, Liu TT, Zhou P, Liu F, Lin X, Tao S, Wan B, Sai-Yin HX, Qin LX, Yin J, Liu L, Wu C, Pei Y, Zhou YF, Zhai Y, Lu PX, Tan A, Zuo XB, Fan J, Chang J, Gu X, Wang NJ, Li Y, Liu YK, Zhai K, Zhang H, Hu Z, Liu J, Yi Q, Xiang Y, Shi R, Ding Q, Zheng W, Shu XO, Mo Z, Shugart YY, Zhang XJ, Zhou G, Shen H, Zheng SL, Xu J, Yu L. Genetic variants in STAT4 and HLA-DQ genes confer risk of hepatitis B virus-related hepatocellular carcinoma. Nature genetics 2013;**45**(1):72-5 doi: 10.1038/ng.2483[published Online First: Epub Date]|.
3. Zheng W, Long J, Gao YT, Li C, Zheng Y, Xiang YB, Wen W, Levy S, Deming SL, Haines JL, Gu K, Fair AM, Cai Q, Lu W, Shu XO. Genome-wide association study identifies a new breast cancer susceptibility locus at 6q25.1. Nature genetics 2009;**41**(3):324-8 doi: 10.1038/ng.318[published Online First: Epub Date]|.
